# Sporozoite motility as a quantitative readout for anti-CSP antibody inhibition

**DOI:** 10.1038/s41598-022-22154-8

**Published:** 2022-10-13

**Authors:** C. M. de Korne, R. van Schuijlenburg, J. C. Sijtsma, H. M. de Bes, E. Baalbergen, S. Azargoshasb, M. N. van Oosterom, M. B. B. McCall, F. W. B. van Leeuwen, M. Roestenberg

**Affiliations:** 1 Leiden University Center for Infectious Diseases (LU-CID), Leiden University Medical Center, Albinusdreef 2, 2333 ZA Leiden, The Netherlands; 2 Interventional Molecular Imaging Laboratory, Department of Radiology, Leiden University Medical Center, Albinusdreef 2, 2333 ZA Leiden, The Netherlands; 3 Department of Medical Microbiology, Radboud University Medical Center, Geert Grooteplein Zuid 10, 6525 GA Nijmegen, The Netherlands

**Keywords:** Parasitology, Vaccines, Infectious diseases, Fluorescence imaging, Time-lapse imaging

## Abstract

Antibodies can prevent malaria by neutralizing the infectious *Plasmodium falciparum* sporozoites (SPZ) before they establish an infection in the liver. Circumsporozoite protein (CSP), the most abundant surface protein of SPZ is the leading candidate for passive (and subunit) immunization approaches against malaria. Comprehensive assessment of the parasite-inhibitory capacity of anti-CSP monoclonal antibodies (mAbs) is an important step in advancing CSP-based immunization strategies. In this study, we employed a quantitative imaging-based motility assay to quantify the effect of anti-CSP mAbs on SPZ motility, both in vitro and in human skin.

Our assay provided a quantitative measure of mAb parasite-inhibitory capacity through measurement of the half-maximal motility inhibitory concentration (IC_50M_) value for anti-CSP mAbs (IC_50M_ 2A10: 24 nM, IC_50M_ 3SP2: 71 nM). We found a sevenfold discrepancy between the IC_50M_ and the binding saturation concentration measured by ELISA, possibly related to the observed shedding of CSP-mAb complexes during SPZ movement. In a subset of SPZ (5%), in vitro motility was unaffected by the presence of 2A10 while 3SP2 was able to completely block movement. In our ex vivo skin explant model, SPZ proved less susceptible to anti-CSP mAbs compared to SPZ in an in vitro environment. By quantitatively assessing motility, we created a valuable tool that can be used for comprehensive assessment of anti-CSP mAb potency. Insight that will help deepen our understanding of anti-CSP mAb potency and guide selection of the most promising anti-CSP mAbs for downstream clinical development.

## Introduction

Since the gains of the current approaches have levelled off and malaria still causes more than 500,000 deaths annually, controlling and eventually eradicating malaria requires additional strategies^1^. Currently, the passive transfer of monoclonal antibodies (mAbs) as a prevention strategy for malaria infections is gaining traction. The concept of passive immunization against malaria is not new^2^, but only recently it has been demonstrated that it can induce protection in humans against a controlled malaria infection and prevent onward transmission^3,4^. Passive immunization can be a valuable approach to induce short-term protection (up to 6 months) against malaria for e.g. pregnant women, military personnel and residents of areas with highly seasonal transmission (e.g. the Sahel sub-region of Africa)^5^.

So far, the target of these anti-malaria infection mAbs (and leading malaria vaccine candidates) is the circumsporozoite protein (CSP), abundantly presented at the sporozoite (SPZ) surface^6^. SPZ are considered an attractive vaccine target, since this early life cycle stage is the longest extracellular stage of the life cycle (ranging from minutes to hours^7,8^). This stage also presents a bottleneck for the infection process as approximately only 1–500 SPZ are inoculated by a mosquito^9,10^. CSP is thought to play a critical role in SPZ migration; an essential process which enables the SPZ to migrate from the inoculation site to the liver and invade hepatocytes to establish a full-blown infection^11,12^. In line with this, in vivo studies suggest that the direct impact of anti-CSP mAbs on the motility of SPZ hampers their dermal exit, thus contributing to the protective effect of anti-CSP mAbs in preventing liver infection^13–16^.

Despite the direct effect of anti-CSP mAbs on SPZ motility, a quantitative assay for this parameter is lacking. Currently, the binding capacity of anti-CSP mAbs for CSP is assessed by ELISA and some of their functional effects on SPZ have been assessed in gliding, cell traversal and hepatocyte invasion assays^17–19^. However, these assays are not sufficiently quantitative to rank the parasite-inhibitory capacity of the increasing number of anti-CSP mAbs being produced nowadays which is needed to select the most potent candidates for downstream (pre)clinical testing.

We hypothesize that an assay capable of more comprehensive quantitative assessment of anti-CSP mAb functionality (including not only binding capacity and hepatocyte invasion inhibition, but also in vitro and in skin motility interference) will aid the identification of efficacious anti-CSP mAbs. As such, we have employed SMOOT (Sporozoite Motility Orienting and Organizing Tool^20,21^), which has been developed to detect subtle differences in SPZ motility, to quantitate the impact of anti-CSP mAbs in an in vitro and ex vivo human skin experimental model. As a proof-of-concept, we studied the functionality of two commonly used and known inhibitory anti-CSP mAbs 2A10^22^ and 3SP2^23^ and explored the coherence between mAb-antigen binding affinity and the mAb’s functional effects on SPZ motility and hepatocyte invasion. Overall, this study established the quantification of SPZ motility as a valuable additional part of the assessment of anti-CSP mAb functionality needed to support the development of CSP-based passive immunization strategies for malaria.

## Methods

### Sporozoite production

*Plasmodium falciparum* (NF54 strain) SPZ were obtained, both wildtype SPZ and transgenic SPZ that express the fluorescent reporter protein GFP under the CSP promotor (NF54 − ΔPf47–5’*csp*-GFP-Luc, kindly provided by dr. Koen Dechering; TropIQ Health Sciences) or the fluorescent reporter protein mCherry under the Sui1 promotor (line Pf-Exp. 159 as described by Miyazaki et al*.*^24^). *Plasmodium falciparum* gametocyte cultures were generated using standard culture conditions in a semi-automated culture system and subsequently gametocytes were fed to *Anopheles stephensi* female mosquitoes by standard membrane feeding as described previously^25,26^. SPZ were obtained by manual dissection of the salivary glands of mosquitoes 14–17 days post infection. The salivary glands were collected and homogenized to release SPZ in Roswell Park Memorial Institute medium (RPMI; Thermo Fisher Scientific) enriched with 10% fetal bovine serum (FBS; Thermo Fisher Scientific) and 1% penicillin–streptomycin (PS; Thermo Fisher Scientific) unless otherwise stated. The free SPZ were counted in a Bürker counting chamber using phase-contrast microscopy and used for experiments immediately or frozen down (> 1 week at − 20 °C) to obtain SPZ lysate for ELISA.

### Whole SPZ ELISA for mAb affinity measurements

Thawed SPZ lysate was suspended to a concentration of 0.2*10^6^ SPZ/ml in phosphate buffered saline (PBS; Thermo Fisher Scientific), plated at 50 µl/well in a flat bottom clear, half-area 96 well plate (Corning) and incubated overnight at 4 °C. The solution was gently removed from the wells and the plate was air dried for 30 min. Plates were then blocked with PBS + 1% FBS for 1 h at room temperature (RT) and washed with PBS + 0.05% Tween 20 (Sigma-Aldrich). To assess the binding affinity of the anti-CSP mAbs 2A10 (obtained through BEI Resources, contributed by prof. E. Nardin) and 3SP2 (^23^; Radboudumc), these mAbs were titrated in threefold serial dilutions (concentration range: 1.0 µg/ml–0.02 ng/ml, control: no mAb) in PBS + 1% FBS and allowed to bind to the SPZ lysate-coated surface for 1 h at RT. After washing, the wells were incubated with a secondary antibody (HRP labelled goat anti-mouse IgG antibody, 1:1000 in PBS + 1% FBS; Invitrogen) for 1 h at RT. After washing, a color reaction was induced using a substrate solution (TMB Microwell Peroxidase Substrate System; KPL) following the manufacturer’s instructions and subsequently stopped by adding stop solution (1.8 M sulphuric acid in MilliQ; Merck). Plates were read on a Multiskan FC Microplate Photometer (Thermo Scientific) at 450 nm absorption. All experiments were performed *in duplo*, containing two samples per concentration per experiment. Competition curves were obtained by plotting the obtained absorbance values against the mAb concentration in logarithmic scale. Sigmoidal curves were fitted to a four-parameter logistic equation using GraphPad Prism (version 9.0.1; GraphPad Software), from which IC_50_ values (concentration at which binding of the mAbs to SPZ lysate was inhibited by 50%) were determined.

### Elution procedure for mAb avidity measurements

To measure the avidity of the anti-CSP mAbs 2A10 and 3SP2, the whole SPZ ELISA was performed as described above with the addition of an elution procedure with ammonium thiocyanate (NH_4_SCN; Sigma-Aldrich) performed between the incubation steps with the primary mAbs (2A10: 10 ng/ml, 3SP2: 100 ng/ml) and the secondary antibody. A concentration range of ammonium thiocyanate in 0.1 M sodium phosphate pH 6.0 was added to the wells (concentration range: 1.0 M–1.0 mM, control: no sodium thiocyanate; Sigma-Aldrich) and incubated for 20 min at RT. The absorbance readings in the presence of increasing concentrations of ammonium thiocyanate were converted to the appropriate percentage of the total bound mAb in the absence of ammonium thiocyanate. For both 2A10 and 3SP2 the avidity index was estimated, representing the molar concentration of thiocyanate required to reduce the initial absorbance by 50%.

### Sporozoite motility in vitro

To assess the effect of the anti-CSP mAbs (2A10 & 3SP2) on in vitro SPZ motility, SPZ expressing mCherry were pre-incubated with a concentration range (control: no antibody, 0.5, 1, 2.5, 5, 10, 25, 50, 100 µg/ml) of the anti-CSP antibodies 2A10 or 3SP2 in RPMI + 10% FBS for 30 min at RT and subsequently (without washing away mAbs) 0.5*10^5^ SPZ were pipetted on the coverslip of a non-coated confocal dish and covered. Movies were recorded on a Leica TCS (true confocal scanning) SP5 microscope (Leica Microsystems, Wetzlar) at 37 °C under 5% CO_2_ conditions using a 561 nm laser to excite mCherry. The emission signal was collected between 600 and 650 nm and movies were recorded with a frame rate of 35 frames per minute, 200 frames per movie. The movies were further processed using SMOOT_In vitro_; in-house developed motility analysis software written in the MATLAB programming environment (The MathWorks Inc.). The movement patterns of the SPZ were classified as floating, stationary or moving (circling) and the velocity of the moving SPZ was determined as previously described^20^. In total, movies from two independent experiments containing 2–3 samples per concentration were included in the analysis; a total number of 4227 SPZ were analysed, on average 235 ± 95 per condition.

### Localization anti-CSP mAbs

Immunohistochemistry was performed to visualize the localization of anti-CSP mAbs. Wildtype SPZ were incubated with a concentration range ( 0.5, 1, 2.5, 5, 10, 25, 50 µg/ml) of the anti-CSP mAbs 2A10 or 3SP2 in RPMI + 10% FBS for 30 min at RT and subsequently 0.5*10^5^ SPZ were added to a confocal dish without any precoating (ø14 mm; MatTek Corporation) and covered with a coverslip (ø12 mm; VWR Avantor). Subsequently, the secondary antibody (goat anti-mouse IgG Alexa Fluor 488, 1:500 in PBS; Invitrogen) was added and incubated for 45 min at RT. The SPZ were counterstained with Cy5-Methyl-Methyl (50 nm; whole body staining) and Hoechst (10 µg/ml; nucleus staining). Finally, the samples were mounted with Prolong Gold Dapi (Invitrogen) and examined using a Leica TCS (true confocal scanning) SP8X WLL (white light laser) microscope (Leica Microsystems) with a 100 × objective (HCX PL APO 100x/1.40–0.70 OIL CS). The Alexa 488 dye conjugated to the secondary antibody was excited at 488 nm (emission: 500–550 nm), Cy5-Methyl-Methyl was excited at 633 nm (emission: 650–700 nm) and Hoechst was excited at 405 nm (emission: 420–470).

### Sporozoite motility in human skin explant

To assess the effect of the anti-CSP mAbs (2A10 & 3SP2) on SPZ movement in human skin explant, SPZ expressing GFP were pre-incubated with a concentration range (control: no mAb, 10, 25, 50, µg/ml) of the anti-CSP mAbs 2A10 or 3SP2 in RPMI + 10% FBS for 30 min at RT. Following pre-incubation, 10 µl mAb solution containing 0.1*10^5^ SPZ (without washing away mAbs) was injected intradermally into human skin explant using a NanoFil needle and syringe (10 µl NanoFil syringe, MicroFil 28G needle; World precision instruments). Human skin explants from 4 different donors were obtained from collaborating surgical centers immediately after surgery. Anonymized human skin explants were obtained from donors undergoing mastectomy after informed consent, approval was obtained by the Commission Medical Ethics (CME) Leiden under number CME: B18-009. All research has been performed according to relevant guidelines/regulations. Immediately after SPZ injection, the injection site was biopsied using a 6 mm biopsy punch (Stiefel), sliced longitudinally through the center, mounted on a microscopy slide. Movies were recorded using an Andor Dragonfly 500 spinning disk confocal on a Leica DMi8 microscope (Oxford Instruments) with a 40 × objective (HCX PL APO 40x/1.30 OIL). The GFP expressed by the SPZ was excited with the 488 nm laser. The movies were recorded with a frame rate of 40 frames per minute, 200 frames per movie. The motility of the moving SPZ was analysed using SMOOT_Human skin_^21^. In total, 1047 SPZ were analysed, on average 150 ± 41 per condition.

### Sporozoite infection assay

HC-04.J7 cells (a human liver cell line kindly provided by prof. R. Dinglasan) were maintained in culture at 37 °C and 5% CO_2_ in culture medium (IMDM (Lonza) supplemented with 10% FBS and 1% PS). One day prior to infection, the cells were harvested in seeding medium (DMEM without glucose (Thermo Fisher Scientific) supplemented with 5% FBS and 1% PS) and seeded at a density of 4 × 10^4^ cells/well in collagen-coated (50 µg/ml, 30 min incubation at RT; Corning) 96-well plates (for PCR samples: plate with Nunclon Delta surface and for imaging samples: Nunc optical-bottom plate with cover glass base; both Thermo Scientific) which resulted in a confluent monolayer at the day of infection. 4–6 h prior to infection, the seeding medium was replaced by infection medium (seeding medium supplemented with glucose (15 mM; Thermo Fisher Scientific) and Gibco insulin-transferrin-selenium solution (1:100; Thermo Fisher Scientific). Wildtype SPZ were collected as described above in infection medium and pre-incubated with a dilution range of anti-CSP mAbs 2A10 and 3SP2 (threefold serial dilutions, concentration range: 50–69 ng/ml in infection medium, control: no mAb) for 30 min at RT. Subsequently, 4 × 10^4^ pre-incubated SPZ were added to the HC-04.J7 cells by replacing the medium of the cells by 50 µl SPZ solution. Unbound mAb was not removed before addition of the SPZ to the hepatocyte culture. As a negative control, 4 × 10^4^ heat-killed SPZ (15 min at 100 °C) were added to the cells. The SPZ were spun down for 3 min at 1200 RPM and the plates were incubated for 3 h at 37 °C and 5% CO_2_. After the incubation period, the cells were washed with culture medium to remove the non-invaded SPZ. The culture medium was refreshed at day 2. At day 4, the imaging samples were fixed with 4% formaldehyde for 20 min at RT and subsequently stained. The PCR-samples were lysed using 100 µl RLT buffer (25 min incubation; Qiagen). The lysed cells were stored at − 80 °C until further use.

### Imaging-based analysis of hepatocyte infection

To assess SPZ infection by microscopy, the fixed HC-04.J7 cells were permeabilized with 0.5% Triton (Thermo Fisher Scientific) in PBS for 20 min and blocked with 10% FBS in PBS for 45 min. After blocking, the primary anti-Hsp70 (1:200; Thermo Fisher Scientific) and anti-GAPDH (1:1000; University of Edinburgh) antibodies were added to the control samples and incubated o/n at 4 °C. Next, the secondary antibodies (anti-rabbit AF594, 1:500 in PBS & anti-mouse AF488, 1:500 in PBS; Invitrogen) were added to all dishes and incubated for 1 h min at RT. Finally, Hoechst 33,342 (1 mg/ml, 1:200; Sigma-Aldrich) was added to the cells and incubated for 30 min at RT. The samples were stored in PBS at 4 °C until imaging. The samples were imaged using an Andor Dragonfly 500 spinning disk confocal on a Leica DMi8 microscope (Oxford Instruments) with a 40 × objective (HCX PL APO 40x/1.30 OIL). Hoechst was excited with the 405 nm laser, AF488 with the 488 nm laser and AF594 with the 561 laser. The Andor imaging software Fusion (Oxford Instruments) and Fiji software were used to generate a single image from the z-stacks (10 Z-slices, total dept 30 μm) obtained with the different lasers.

### PCR-based analysis of hepatocyte infection

To assess SPZ infection by PCR, the 18S ribosomal RNA (rRNA) levels were determined. To this end, RNA was isolated from the cell cultures using the RNeasy kit (Qiagen) and cDNA was generated using random hexamer primers (Promega) and Superscript™ III (Thermo Fisher) according to manufacturer’s instructions. Amplification reactions of each cDNA sample were performed in PCR plates (hard-shell PCR plate; Bio-Rad), in a volume of 10 μl containing 5 μl PCR buffer (GoTaq qPCR master mix; Promega), 1 μl RNase-free milliQ water, 2 μl primer mix (forward primer: Plasmo Plu3F 5’-GCTCTTTCTTGATTTCTTGGATG-3’ (0.2 µM; Integrated DNA Technologies, Inc.); reverse primer: Plasmo Plu3R 5’- AGCAGGTTAAGATCTCGTTCG-3’ (0.2 µM; Integrated DNA Technologies, Inc.)) and 2 μl of the cDNA sample. Amplification was performed 10 min at 95 °C followed by 50 cycles of 10 s at 94 °C, 15 s at 60 °C, and 30 s at 72 °C. Amplification, detection, and analysis were performed with the CFX96TM real time PCR detection system (Bio-Rad).

### Statistical analysis

Data were summarized using the mean and standard deviation (SD) for parametric data or the median and interquartile range (IQR) for nonparametric data. For the comparison of groups, the difference between means or medians was assessed using respectively the independent sample T test and the Mann–Whitney U test. All statistical tests were performed by SPSS Statistics (IBM Nederland BV). The probability distribution of SPZ velocity was obtained by calculating the probabilities of velocities between 0 and 5 µm/s using the probability density function available in the open source R environment^27^.

## Results

### Binding of anti-CSP mAbs to SPZ

**Figure 1:**
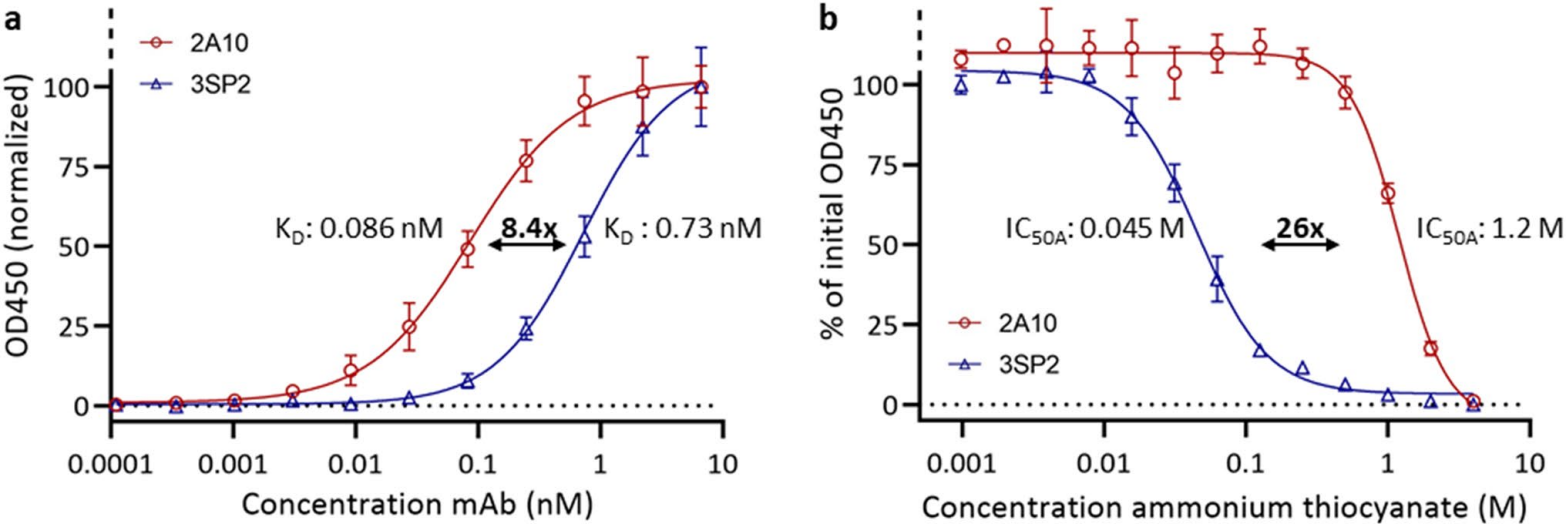
Anti-CSP mAb reactivity to whole SPZ lysate. (**a**) ELISA curve of anti-CSP mAbs binding at different concentrations to whole SPZ lysate to determine mAb affinity (expressed as K_D_). (**b**) ELISA curve of the effect of a serial dilution of ammonium thiocyanate on anti-CSP mAbs binding to whole SPZ lysate to determine mAb avidity (expressed as IC_50A_).

A whole SPZ ELISA was used to confirm the picomolar affinity of the anti-CSP monoclonal antibodies (mAbs) 2A10 and 3SP2 for SPZ. The equilibrium dissociation constant (K_D_) of 2A10 was 8.4-fold lower than the K_D_ of 3SP2 (K_D_ 2A10: 0.086 nM, K_D_ 3SP2: 0.73 nM) (Fig. 1a). These values are in line with those previously reported for anti-CSP mAbs^18^. Saturation of binding sites occurred around 1 nM for 2A10 and 10 nM for 3SP2. The mAbs avidity was determined using thiocyanate elution. The avidity index (IC_50A_: amount of thiocyanate required to reduce antibody-antigen binding by 50%) of 2A10 was 26-fold higher than the avidity index of 3SP2 (IC_50A_ 2A10: 1.2 M, IC_50A_ 3SP2: 0.045 M) (Fig. 1b).

### Effect of anti-CSP mAbs on SPZ behaviour in vitro

**Figure 2:**
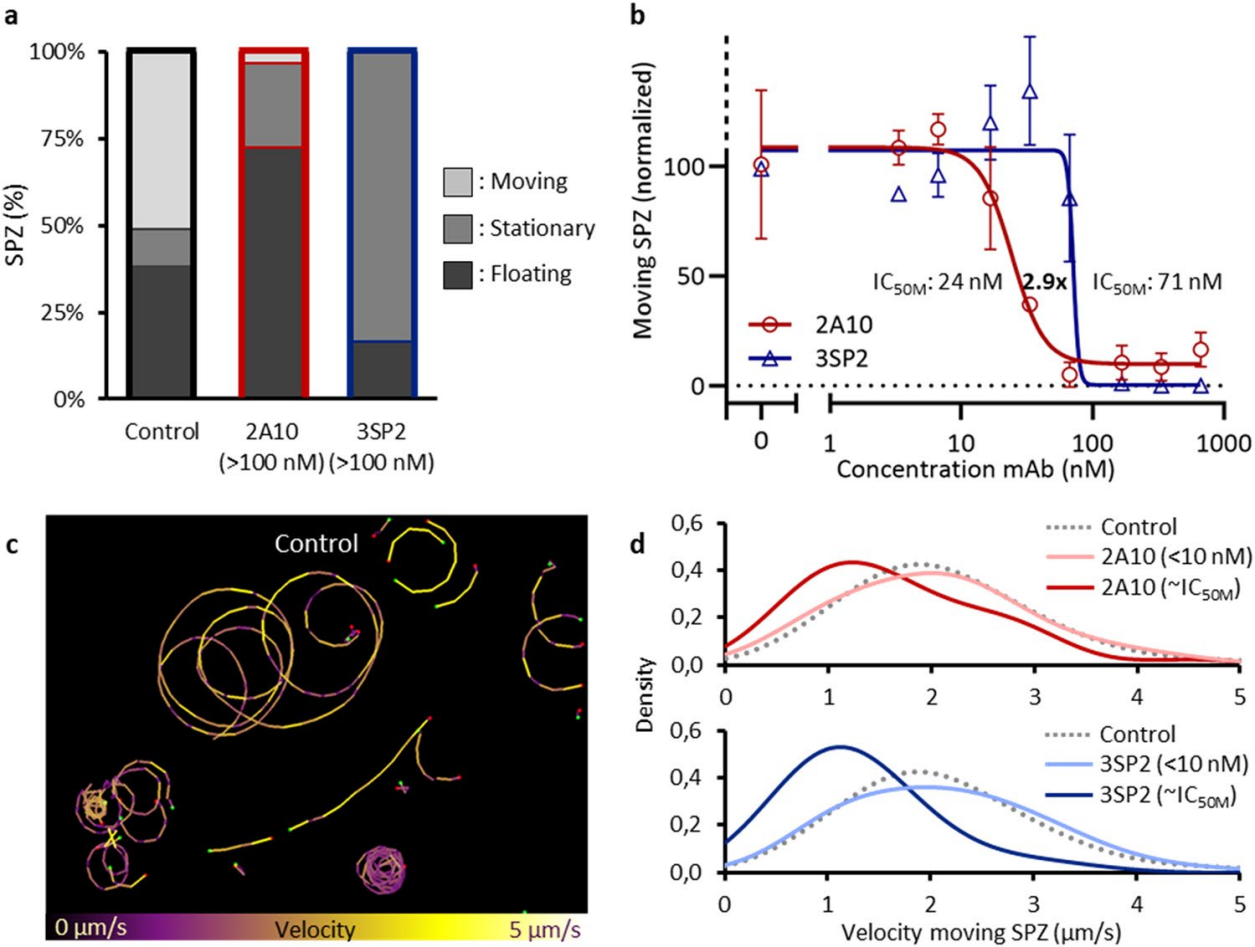
Effect of anti-CSP mAb on SPZ movement and attachment. (**b**) The movement pattern distribution for control SPZ and SPZ exposed to a high concentration (> 100 nM) of the anti-CSP mAbs 2A10 and 3SP2. (**b**) The normalized percentage of moving SPZ plotted against the concentration of mAb to determine the IC_50M_ value at which movement of SPZ was 50% reduced. (**c**) Tracks of SPZ moving in vitro on a glass surface, color-coded for velocity using color range: purple (low velocity) to yellow (high velocity). (**d**) The probability distribution plotted of the velocity of control SPZ (median: 2.0 (IQR: 1.5–2.6) μm/s), SPZ exposed to low concentrations of 2A10 and 3SP2 (7 nM) (median velocity 2A10: 2.1 (IQR: 1.4–2.6) μm/s, median velocity 3SP2: 2.1 (IQR: 1.4–2.8) μm/s ) and SPZ exposed to approximately their IC_50M_ concentration (median velocity while exposed to 33 nM 2A10: 1.4 (IQR: 1.0–2.2) μm/s, median velocity while exposed to 66 nM 3SP2: 1.1 (IQR: 0.9–1.6) μm/s).

To assess the functional effect of the anti-CSP mAbs 2A10 and 3SP2 on SPZ, SPZ were preincubated with the mAbs after which their motility was assessed in vitro. Three movement patterns were discerned: floating, stationary and moving. Roughly 50% of non-exposed control SPZ were moving, about 10% was attached to the surface but not moving and the remaining 40% were floating (Fig. 2a). Interestingly, neither mAb had a detectable effect on SPZ motility at the concentration that fully saturated the available binding sites in a SPZ ELISA (1 nM for 2A10, 10 nM for 3SP2; Fig. 2b). At higher concentrations, the anti-CSP mAbs interfered both with SPZ attachment and motility. Exposure to a concentration of 15 nM 2A10 or higher interfered with attachment of SPZ to the glass surface, which resulted in an increased number of floating SPZ (> 70%) and a reciprocal decreased percentage of moving SPZ (± 5%). In contrast, exposure to 65 nM or higher concentrations of 3SP2 resulted in attached, but non-moving SPZ (stationary: > 80%, moving: 0%) (Fig. 2b). This indicates that two different mechanisms are at play; 2A10 reduces movement mainly through a reduction in surface attachment, while 3SP2 reduces movement by increasing surface attachment and thereby blocking movement. To reduce the percentage of moving SPZ by 50%, threefold more 3SP2 was needed compared to 2A10 (half maximal motility inhibitory concentration (IC_50M_) 2A10: 24 nM, IC_50M_ 3SP2: 71 nM) (Fig. 2b). That said, 3SP2 was able to completely block movement, while even at high concentrations of 2A10 (> 100 nM) 5% of the SPZ continued to move (Fig. 2b).

The impact of the mAbs on the median velocity of the moving SPZ was assessed next. At lower concentrations of mAbs (< 10 nM), SPZ velocity was not reduced compared to untreated controls (2.0 (IQR 1.5–2.6) µm/s; Fig. 2c,d). At concentrations around the IC_50M_ (when roughly half of the SPZ still move; Fig. 2b), both mAbs slowed down the SPZ that were still able to move (median velocity while exposed to 33 nM 2A10: 1.4 (IQR: 1.0–2.2) μm/s, median velocity while exposed to 66 nM 3SP2: 1.1 (IQR: 0.9–1.6) μm/s) (Fig. 2d). At increasing concentrations of 2A10, the SPZ velocity distribution changed shape, indicative for a subpopulation of SPZ less susceptible to the effect of 2A10 (Fig. 2d), which correlated with the abovementioned finding that a small percentage of SPZ continues moving at high concentrations of 2A10. In summary, the functional in vitro motility assessment of mAbs revealed that mAb concentrations above the saturating concentration (as determined by ELISA) are needed to inhibit SPZ motility, mAb motility inhibition mechanisms may vary and that subsets of SPZ may be refractory to the effect of mAbs.

### Localization of anti-CSP mAbs

**Figure 3:**
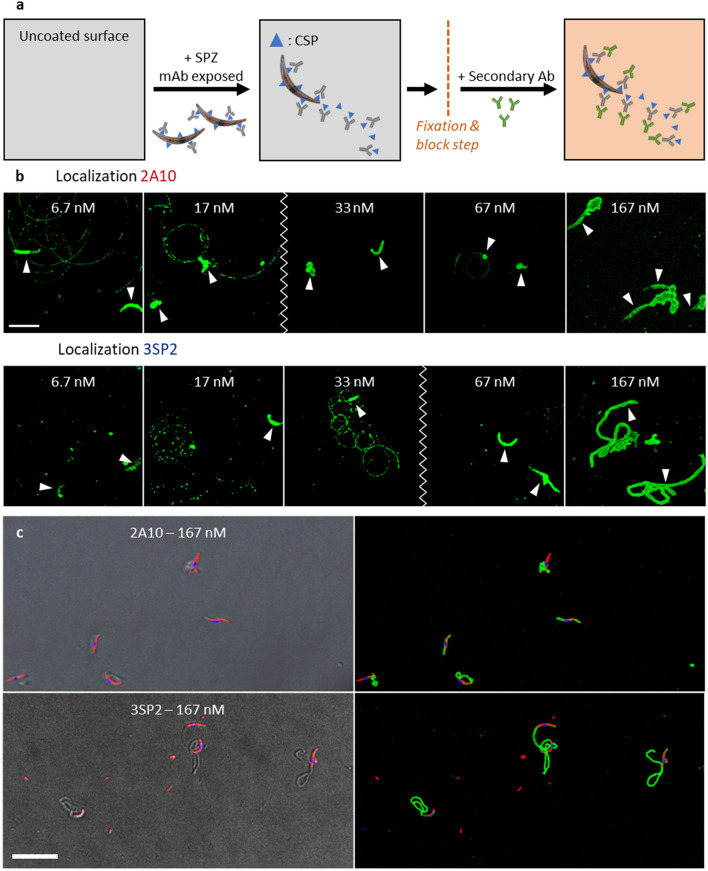
Localization of anti-CSP mAb (**a**) Schematic overview of the localization of bound anti-CSP mAbs using a fluorescent secondary Ab. (**b**) Visualization of the presence of the anti-CSP mAbs 2A10 and 3SP2; the mAbs staining is shown in green, the location of the SPZ is depicted with an arrow and the zigzag line indicates at which concentration the mAbs started to induce protrusions. Scalebar: 10 μm. (**c**) Visualization of the presence and the effect of 2A10 and 3PS2 on SPZ; to the left an overlay of the brightfield image with the SPZ counterstained with Cy5-Methyl-Methyl (shown in red) and Hoechst (shown in blue) and to the right an overlay of the mAbs staining (shown in green) and the counterstained SPZ. Scalebar: 20 µm.

The discrepancy between the mAbs concentrations needed to interfere with movement and those saturating available binding sites based on SPZ ELISA, suggests that mAbs detach from the SPZ during kinematic analysis. To investigate this, we visualized anti-CSP mAbs with a fluorescent secondary Ab staining (Fig. 3a). At all concentrations, the mAbs were located on the SPZ surface (Fig. 3b). At the lower concentrations of mAbs at which the SPZ were still able to move (Fig. 2b), fluorescent spots/trails were visible, indicative of mAbs shedding (Fig. 3b). At concentrations of mAbs that interfered with SPZ attachment and motility, 2A10 (≥ 33 nM) and 3SP2 (≥ 67 nM) were located on protrusions which seemed distinctive for the two mAbs (2A10: elongated, tubular shaped; 3SP2: short, irregularly shaped) (Fig. 3b,c). This indicates that viable SPZ can shed mAbs off their surface.

### Effect of anti-CSP mAbs on SPZ migration patterns in human skin explant

**Figure 4:**
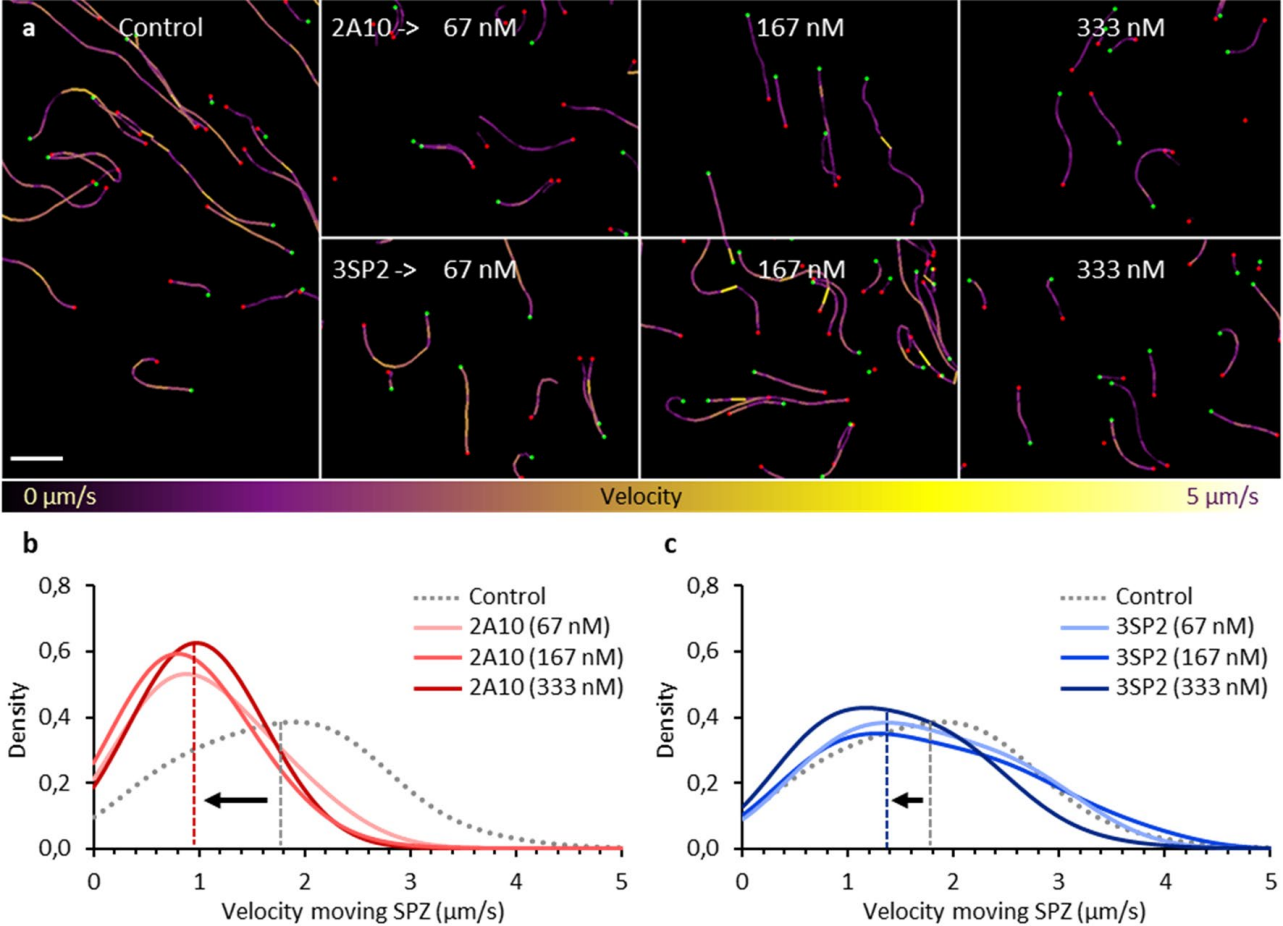
SPZ velocity in human skin explant (**a**) Tracks of SPZ moving in human skin explant, color-coded for velocity using colour range: purple (low velocity) to yellow (high velocity). Scalebar: 100 µm. (**b**) The probability distribution plotted of the velocity of control SPZ (median: 1.8 (IQR: 1.1–2.3) μm/s, median annotated with grey dashed line) and SPZ exposed to increasing concentrations (67, 167, 333 nM) of 2A10 (median velocity SPZ exposed to 333 nM: 1.0 (IQR: 0.7–1.3) μm/s, median annotated with red dashed line). (**c**) The probability distribution plotted of the velocity of control SPZ and SPZ exposed to increasing concentrations (67, 167, 333 nM) of 3SP2 (median velocity SPZ exposed to 333 nM: 1.4 (IQR: 0.8–2.0) μm/s, median annotated with blue dashed line).

Next, we set out to compare the effect of 2A10 and 3SP2 on SPZ migration patterns in human skin explant. As reported, SPZ migration is markedly different in the 3D skin environment, with more ‘directional’ linear tracks, as compared to the ‘default’ circular movement which SPZ exhibit *in vitro*^21^ (Fig. 4a). Contrasting the mAb concentrations needed in vitro to impact SPZ motility described above, pre-incubation with 2A10 and 3SP2 up to concentrations of 333 nM for 30 min did not block SPZ motility as compared to untreated controls (median velocity of 1.8 (IQR: 1.1–2.4) μm/s (Fig. 4b,c)). Only at high concentrations (≥ 67 nM for 2A10 and 333 nM for 3SP2) the SPZ velocity significantly decreased to respectively 1.0 (IQR: 0.6–1.5) and 1.4 (IQR: 0.8–2.0) μm/s (*p* < 0.001 and *p* = 0.002, Mann–Whitney U test) (Fig. 4b,c). Increasing concentrations of 2A10 did not further reduce SPZ velocity (median velocity SPZ exposed to 333 nM 1.0 (IQR: 0.7–1.3) μm/s; p = 0.829 when compared to 67 nM, Mann–Whitney U test) which corresponded with the abovementioned finding that a subset of SPZ is less susceptible to the effect of 2A10. Altogether, these results indicate that ex vivo higher concentrations of mAbs are needed to inhibit motility as compared to the in vitro assay.

### Effect of anti-CSP mAbs on SPZ infectivity

**Figure 5:**
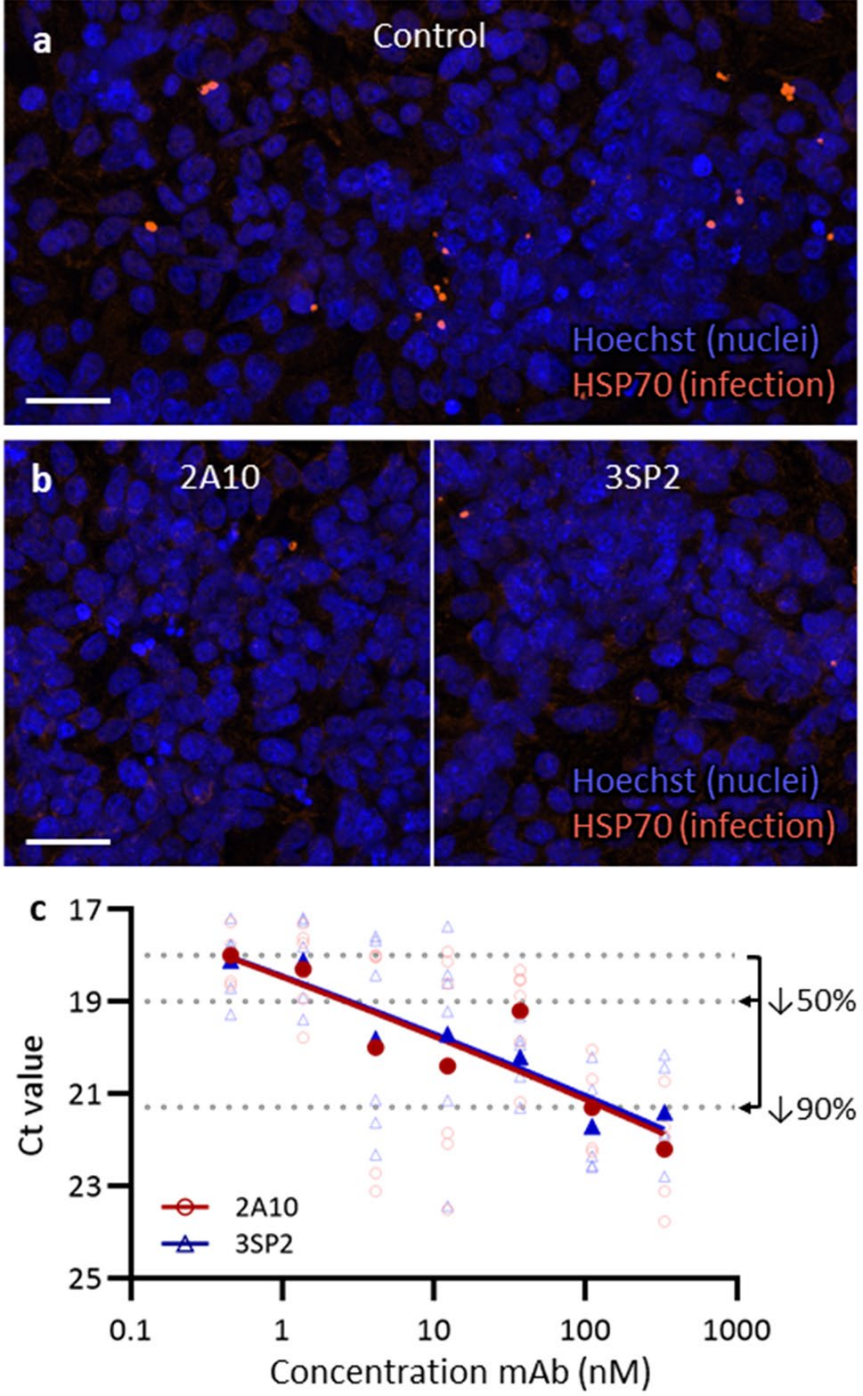
Effect of anti-CSP mAb on HC-04.J7 infection rate (**a**) Immunofluorescent staining of schizonts in HC-04.J7 cells by anti-Hsp70 staining (orange) and counterstaining with Hoechst (blue). Scale bar: 30 µm. (**b**) Images of the liver stage development assay representative for exposure to 333 nM 2A10 and 3SP2. Fluorescent anti-Hsp70 staining (orange) and counterstaining with Hoechst (blue). Scale bar: 30 µm. (**c**) The Ct (cycle threshold) value of 6 wells (open symbols) and the median (closed symbols) obtained by RT-PCR plotted against the concentration of mAb. The Ct values that represent a decrease of 50% and 90% in liver stage development are annotated.

The functional effect of the anti-CSP mAbs on SPZ invasion of hepatocytes was assessed using the HC-04.J7 hepatocyte cell line. Control SPZ readily infected the hepatocytes, which was confirmed by microscopy using an anti-Hsp70 staining (Fig. 5a). Exposure to 2A10 and 3SP2 inhibited hepatocyte invasion (Fig. 5b), which was quantified using RT-PCR. A dose-dependent reduction in invasion was found for both anti-CSP mAbs, despite high assay variability (Fig. 5c). Exposure to low concentrations of 4.1 nM of both 2A10 and 3SP2 mAb reduced the hepatocyte infection by ≥ 50%. No difference in the potency of 2A10 and 3SP2 to inhibit hepatocyte infection was observed.

## Discussion

We were able to establish a quantitative motility assay that enables assessment of anti-CSP mAbs inhibitory capacity against *Plasmodium falciparum (Pf)* SPZ. Interestingly, anti-CSP mAb concentrations far above the saturating binding concentration (as determined by ELISA) were needed to inhibit SPZ motility in vitro and these values increased even further in human skin explants*.* In addition, we found that motility inhibition differs between anti-CSP mAbs and that subsets of SPZ can be refractory to the effect of mAbs. These findings underline that a comprehensive assessment of anti-CSP mAb functionality, including SPZ motility inhibition, is needed to characterize their parasite-inhibitory efficacy and predict potency.

Real-time tracking of SPZ combined with the SPZ motility analysis tool SMOOT enabled us to estimate a half-maximal motility inhibitory concentration (IC_50M_) for both mAbs, providing a quantitative measure of mAb functionality. So far, *Pf* SPZ motility in vitro in the presence of a mixture of antibodies has only been assessed post hoc by staining and subsequent counting of the trails produced by gliding SPZ, a qualitative rather than quantitative analysis subject to considerable assay variability^17,24^. SMOOT was able to pick up subtle differences in SPZ motility, ultimately deepening our understanding of anti-CSP mAb functionality. We found a remarkable discrepancy between the IC_50M_ and the binding saturation concentration measured by ELISA, which may be caused by shedding of mAbs by viable SPZ during shedding and replenishing CSP^28^. Exposure to anti-CSP mAbs has previously been suggested to enhance the secretion of CSP^13,29,30^. This could mean that the functionality of anti-CSP mAbs does not merely rely on their binding affinity and avidity, but also on their impact on the CSP shedding rate. This may explain why a direct correlation between binding capacity of anti-CSP mAbs and their inhibitory activity in in vitro and in vivo models is lacking^31^.

In this study we are the first to assess anti-CSP mAb functionality in human skin*.* SPZ exhibit more directional movement in their natural skin environment compared to the mere circular movement on a 2D surface. Although mAbs reduced the velocity of SPZ migrating through skin, they did not block SPZ motility to the same extent as observed in vitro. This is in line with the reduced motility reported in studies investigating SPZ in the skin of passively immunized mice^13,14,16,32^. It remains to be determined why SPZ are relatively refractory to the effects of anti-CSP mAbs in skin. Potentially, redundancy in binding sites for CSP or the molecular mechanisms regulating SPZ motility in their natural environment may be part of the explanation.

Hitherto, functional assays to investigate the effect of anti-CSP mAbs on SPZ have mainly focused on the liver stage of the infection by measuring the inhibition of hepatocyte traversal/invasion or liver stage development (ILSDA)^33–37^. Nevertheless, mice studies have suggested that immobilizing SPZ after intradermal inoculation would likely limit their chances to reach and infect the liver^38^. In our study, both the results from the motility analysis and the ILSDA showed a dose dependent inhibitory effect of anti-CSP mAbs on SPZ, however only the former revealed differences between anti-CSP mAbs. This incoherence may have arisen from different mechanisms involved in motility and hepatocyte invasion, that were unequally susceptible to anti-CSP mAb inhibition. The differences may also have been masked in the ILSDA due to the high assay variability^33,36,39^). Either way, these findings underline that a motility-based functional assay provides valuable complementary information about the parasite-inhibitory potency of anti-CSP mAbs.

The anti-CSP mAbs 2A10 and 3SP2 differed in the way they interfered with motility in vitro, whereby 2A10 reduced attachment to the surface, while 3SP2 increased attachment. Remarkably, only 3SP2 could block motility completely. Other studies have also suggested that the direct effect of anti-CSP mAbs on SPZ is mediated by different mechanisms of action, dependent on their binding capacity^31^, the recognized epitope(s)^13,19,40,41^ and the possibility to crosslink CSP or otherwise affect CSP conformation^30,42,43^. Assessing which epitope(s) are recognized by 2A10 and 3SP2 and how they affect CSP confirmation would help to further elucidate the mechanistic basis of the found differences. Especially the finding that 2A10, despite its higher binding capacity, could not block SPZ movement may have consequences for the potency of mAbs targeting a similar CSP epitope.

Despite being technically more challenging, extending assessment of anti-CSP mAbs potency by measuring their motility inhibitory capacity can help to select the most promising candidates for passive immunization against malaria^3,44^. Passive transfer of mAbs is a strategy which bypasses obstacles encountered during active immunization strategies, such as immunoregulatory effects of previous malaria exposure and the influence of age on immune responses^5^. To extend the short-term protection (up to 6 months) possibly induced by passive immunization, mAbs could potentially be delivered via a viral vector; an approach which has shown potential for protecting against malaria and other infectious diseases in preclinical models^45,46^. In addition, selection of high potential mAbs for epitope characterization can provide a means to support the rational design of subunit vaccines^6^. For example, an epitope within the junctional region of CSP was recently identified as a target of mAbs with protective efficacy and may become part of the sequence of next-generation CSP-based subunit vaccines^47^. Future research would be needed to develop a prediction model that includes the results, weighted based on their discriminative power, of different assays to score and rank anti-CSP mAbs potency which may aid more accurate identification of potential candidates for downstream (pre)clinical testing.

Taken together, our motility-based assay SMOOT can support comprehensive functionality assessment of anti-CSP mAbs which recently became available and which will continue to appear^18,19,31,39,44^. Thus deepening our understanding of anti-CSP mAb functionality and aiding the selection of lead candidates for passive immunization strategies against malaria.

## Data Availability

The data can be made available upon request by contacting the corresponding author (M.Roestenberg@lumc.nl).
